# Insights into the Superrosids phylogeny and flavonoid synthesis from the telomere-to-telomere gap-free genome assembly of *Penthorum chinense* Pursh

**DOI:** 10.1093/hr/uhad274

**Published:** 2023-12-19

**Authors:** Zhoutao Wang, Junmei Zhou, Junjie Pan, Wei Cheng, Jie Fang, Qundan Lv, Xiaodan Lin, Wenliang Cheng, Liangsheng Zhang, Kejun Cheng

**Affiliations:** Genomics and Genetic Engineering Laboratory of Ornamental Plants, College of Agriculture and Biotechnology, Zhejiang University, Hangzhou 311300, China; Postdoctoral Research Enter, Zhejiang Kangning Pharmaceutical Co., Ltd, Lishui 323000, China; Product Development Department, Zhejiang Shaowei Yuanzhi Science and Technology Development Co., Ltd, Lishui 323000, China; Postdoctoral Research Enter, Zhejiang Kangning Pharmaceutical Co., Ltd, Lishui 323000, China; Postdoctoral Research Enter, Zhejiang Kangning Pharmaceutical Co., Ltd, Lishui 323000, China; Genomics and Genetic Engineering Laboratory of Ornamental Plants, College of Agriculture and Biotechnology, Zhejiang University, Hangzhou 311300, China; Postdoctoral Research Enter, Zhejiang Kangning Pharmaceutical Co., Ltd, Lishui 323000, China; Postdoctoral Research Enter, Zhejiang Kangning Pharmaceutical Co., Ltd, Lishui 323000, China; Postdoctoral Research Enter, Zhejiang Kangning Pharmaceutical Co., Ltd, Lishui 323000, China; Zhejiang Provincial Key Laboratory of Resources Protection and Innovation of Traditional Chinese Medicine, Zhejiang A&F University, Hangzhou, 311300, China; Postdoctoral Research Enter, Zhejiang Kangning Pharmaceutical Co., Ltd, Lishui 323000, China; Genomics and Genetic Engineering Laboratory of Ornamental Plants, College of Agriculture and Biotechnology, Zhejiang University, Hangzhou 311300, China; Postdoctoral Research Enter, Zhejiang Kangning Pharmaceutical Co., Ltd, Lishui 323000, China; Product Development Department, Zhejiang Shaowei Yuanzhi Science and Technology Development Co., Ltd, Lishui 323000, China; Zhejiang Provincial Key Laboratory of Resources Protection and Innovation of Traditional Chinese Medicine, Zhejiang A&F University, Hangzhou, 311300, China

## Abstract

The completion of the first telomere-to-telomere (T2T) genome assembly of *Penthorum chinense* Pursh (PC), a prominent medicinal plant in China, represents a significant achievement. This assembly spans a length of 257.5 Mb and consists of nine chromosomes. PC’s notably smaller genome size in Saxifragales, compared to that of *Paeonia ostii*, can be attributed to the low abundance of transposable elements. By utilizing single-copy genes from 30 species, including 28 other Superrosids species, we successfully resolved a previously debated Superrosids phylogeny. Our findings unveiled Saxifragales as the sister group to the core rosids, with both being the sister group to Vitales. Utilizing previously characterized cytochrome P450 (CYP) genes, we predicted the compound classes that most CYP genes of PC are involved in synthesizing, providing insight into PC’s potential metabolic diversity. Metabolomic and transcriptomic data revealed that the richest sources of the three most noteworthy medicinal components in PC are young leaves and flowers. We also observed higher activity of upstream genes in the flavonoid synthesis pathway in these plant parts. Additionally, through weighted gene co-expression network analysis, we identified gene regulatory networks associated with the three medicinal components. Overall, these findings deepen our understanding of PC, opening new avenues for further research and exploration.

## Introduction

*Penthorum chinense* Pursh (PC), commonly known as ‘GanHuang Cao’ in traditional Chinese medicine, has been used by the Miao nationality for centuries [1, 2]. Numerous previous studies have confirmed the various health benefits associated with PC extract, including anti-inflammatory, hepatoprotective, and antioxidant effects [3–6]. One of the primary therapeutic effects is its ability to protect the liver by reducing the inflammatory response and preventing the entry of oxygen-free radicals [7–9]. The main components found in PC’s phytochemical profile are flavonoids [10–12]. Several epidemiological and clinical investigations have consistently demonstrated that flavonoids exhibit strong antioxidant properties *in vivo* and *in vitro*, as well as anti-inflammatory, anticancer, antiobesity, antidiabetic, and antibacterial activities [13–15]. Infrared spectroscopy has provided evidence demonstrating that PC contains a significant amount of kaempferol, quercetin, and pinocembroside, which produced liver-protective effects, antioxidant, and anti-inflammation [10–12].

Despite notable advancements in pharmacological research on PC [1, 2, 7–9], there have been limited studies to comprehend the molecular mechanism responsible for synthesizing its primary active ingredients, especially the flavonoids as mentioned previously. Through the integration of transcriptomics and metabolomics, we can gain rich insights into the synthesis mechanisms of those active ingredients in PC. Additionally, the plant cytochrome P450 (CYP) families are dynamically diverse across taxa, leading to variations in gene families, subfamilies, and their proportions, which result in notable qualitative and quantitative variability in metabolite profiles [16–18]. Studying the expression, evolution, and distribution of CYP genes can also provide important insights into the synthesis mechanisms of primary active ingredients in PC. Unfortunately, due to the absence of a reference genome, these essential studies have not been conducted in PC.

Solving the deep-level relationships within the Superrosids clade presents significant challenges due to its complexity. This clade includes approximately 25% of all angiosperms and is comprised of 18 orders. These orders can be further classified into three subclades: Vitales, Saxifragales, and the core rosids. However, previous studies have yielded three inconsistent scenarios regarding the phylogenetic relationships among the three subclades [19]. Some studies propose that Saxifragales are sister to the Vitales and together form the sister group to the core rosids [20, 21]. Alternatively, some studies suggest that Saxifragales are sister to Vitales + core rosids [22, 23]. Lastly, other studies indicate that Saxifragales are sister to the core rosids, with both being sisters to Vitales [24, 25]. Currently, there has been a notable increase in the availability of genomic data for species belonging to the Saxifragales, Vitales, and the core rosids. This growing dataset enables more accurate determination of phylogenetic relationships by analysing the entire genome landscape. By leveraging this comprehensive dataset, researchers can gain improved insights into the relationships within the Superrosids clade.

The first primary objective of this study is to construct a T2T-level genome assembly for PC, which will be the first T2T-level assembly in Saxifragales. Subsequently, by combining this assembly with genomic information from other sequenced species in Saxifragales and Vitales, we aim to establish a more comprehensive understanding of the evolutionary relationships within the three subclades of the Superrosids clade. Furthermore, by predicting the functions of the genome-wide CYP superfamily, an insight will be gained into the potential metabolic diversity of PC. Lastly, this study will provide a valuable reference for the tissue-specific distribution characteristics and synthesis regulatory mechanisms of the three most noteworthy medicinal components in PC through large-scale transcriptome and metabolome analysis.

## Results

### Genome assembly, annotation, and evaluation

**Figure 1:**
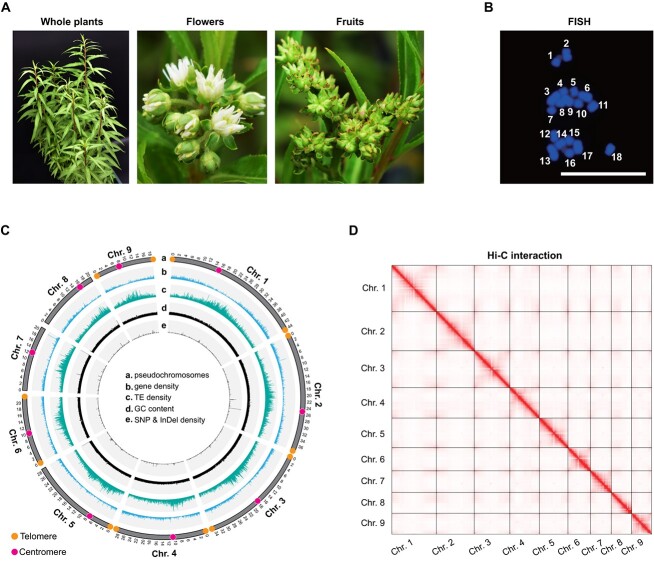
Genomic characteristics of *Penthorum chinense* Pursh (PC) are shown. **A** Whole plants, flowers, and fruits of PC*.***B** Fluorescence *in situ* hybridization (FISH) analysis demonstrates that the haploid genome of PC has nine chromosomes. **C** Tracks from outside to inside show nine chromosomes (a), gene density (b), transposable element (TE) density (c), GC content (d), density of single nucleotide polymorphisms (SNPs) and insertion and deletion polymorphisms (InDels) (e). The locations of telomeres and centromeres have been labeled in the diagram, respectively. The densities were calculated with 50 kb sliding windows. **D** Hi-C interaction heat map between the nine chromosomes for the PC genome.

The PC (Fig. 1A) genome was estimated to be approximately 257.2 Mb with a relatively low heterozygosity of 0.24% (Fig. S1A, see online supplementary material). This estimation was based on the analysis of *k*-mer frequencies (*k* = 19) from Illumina short reads. Additionally, flow cytometry experiments yielded an average haploid genome size estimate of 263.6 Mb (Table S1, see online supplementary material), which closely matched the estimation obtained from *k*-mer analysis. Fluorescence *in situ* hybridization revealed that the haploid genome of this species consists of nine chromosomes (Fig. 1B).

The PC genome was assembled using a combination of 17.2 Gb (∼67.0×) of filtered ONT, 15.6 Gb (∼60.7×) of Illumina, and 109.6 Gb (∼426.6×) of Hi-C data. The initial assembly consisted of 15 contigs (N50 = 28.7 Mb). Employing Hi-C data, 99.7% of the bases were connected to form nine chromosomes (Fig. 1C and D). After linking contigs, two gaps were generated and subsequently repaired. Finally, the Hi-C interaction indicates that the gap-free assembly, with a size of 257.5 Mb, had no obvious assembly errors and perfectly comprised nine clusters at the chromosomal level (Fig. 1D). The GC depth graph demonstrates that the assembly was free from other species’ biological DNA sequences (Fig. S1B, see online supplementary material). The statistics of the genome assembly and annotation were presented in detail in Table 1.

**Table 1:** Statistics of genome assembly and annotation of *Penthorum chinense* Pursh.

**Type**	**Parameter**	**Value**
Assembly	Estimated genome size by flow cytometry (Mb)	263.6
	Estimated genome size by *k*-mer frequencies (Mb)	257.2
	Total assembly size (Mb)	257.5
	No. of chromosomes	9
	No. of unconnected contigs	4
	No. of gaps	0
	Viridiplantae BUSCO coverage (%)	99.7
	GC content (%)	34.2
Annotation	No. of genes	24 617
	LTR assembly index (LAI)	12.7
	Viridiplantae BUSCO coverage (%)	99.3
	Transposable element content (%)	21.0
	No. of simple sequence repeats (SSRs)	77 898

The telomeres were detected at both ends of Chromosomes (Chr.) 1, 2, 3, 4, 6, and 9 in the assembly. However, for Chr. 5, telomeres were found only at one end, while Chr. 7 and 8 did not show any telomere signals. This suggests that our assembly has achieved a level similar to the T2T standard. Simultaneously, the positions of the centromeres of all chromosomes were successfully predicted, and the reliability of the predictions was confirmed from the low gene density and higher transposable element (TE) density surrounding each predicted centromere position (Fig. 1C).

To assess the completeness of the assembled genome, various additional analyses were conducted. Firstly, approximately 97.5% of the Illumina reads were mapped to the assembly, indicating a high mapping rate. Secondly, the Benchmarking Universal Single-Copy Orthologs (BUSCO) evaluation results (99.7%) obtained from the Viridiplantae databases indicated that the assembly integrity was very satisfactory. Additionally, the LTR assembly index (LAI) [26] also reached an excellent value of 12.7. Using RNA-seq data, we predicted a total of 24 617 gene models with an average length of 2667.7 bp in the assembly. The high complete BUSCO coverage confirmed the accuracy and integrity of our gene annotations (Fig. S1C, see online supplementary material).

After the species differentiation between the PC and *Paeonia ostii*, both belonging to Saxifragales, it is possible that the PC underwent a whole-genome duplication event (Fig. S2A and B, see online supplementary material). However, the *P. ostii* did not undergo a whole-genome duplication. So, why is the *P. ostii*’s genome much larger than that of the PC (Fig. S2C, see online supplementary material)? A reasonable explanation is that the *P. ostii*’s genome has experienced a significant amount of TE replication, with TEs accounting for 81% of its genome (Fig. S2C and D; Table S2, see online supplementary material). On the other hand, the PC and other species within Saxifragales that have similar genome sizes to PC exhibit a much lower proportion of TEs in their genomes.

### Phylogenetic position of Saxifragales in Superrosids

**Figure 2:**
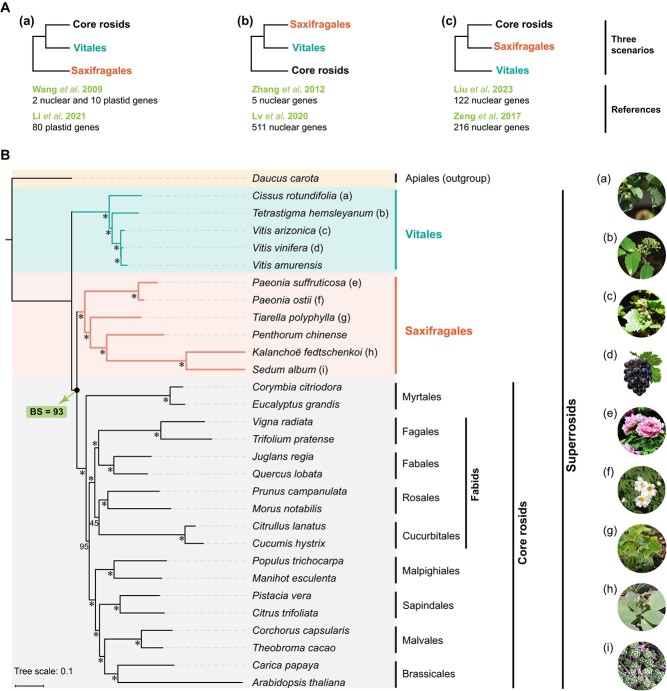
The phylogenetic relationships of three subclades in Superrosids. **A** Three reported topologies of phylogenetic relationships in Superrosids: (a) Saxifragales to be sister to Vitales + core rosids [22, 23]; (b) or sister to Vitales and together as sister to the core rosids [20, 21]; (c) or sister to the core rosids and together as sister to Vitales [24, 25]. **B** Phylogenetic tree generated from a maximum-likelihood analysis of a concatenated protein sequence alignment of 377 single-copy nuclear genes from 30 species. The numbers at each node represent the percent bootstrap support (BS) values from the maximum likelihood analysis, and the asterisk (*) indicates that the clade is supported by a BS value of 100.

This study aimed to investigate the phylogenetic relationships between the three subclades: Saxifragales, Vitales, and core rosids within Superrosids. Previous studies have presented conflicting scenarios [20–25], highlighting the need for further examination. Please refer to Fig. 2A for an illustration of the three conflicting scenarios. To address this, we conducted a genome-based phylogeny analysis of PC and 28 other species belonging to the Superrosids clade. Initially, employing 377 single-copy genes, a phylogeny was inferred using the Maximum Likelihood (ML) method with *Daucus carota* serving as the outgroup. The resulting phylogeny (Fig. 2B) strongly supported topology ‘c’ as depicted in Fig. 2A. It robustly concluded that Saxifragales is the sister group to the core rosids [bootstrap support (BS) = 93] and that both of them are the sister group to Vitales.

To enhance the reliability of our conclusion, we made two modifications in our phylogeny analysis. Firstly, we substituted the outgroup species with *Angelica sinensis* (Apiaceae). Additionally, we excluded certain species within Vitales and Saxifragales that belonged to the same genus, as well as species within the core rosids that belonged to the same order. These adjustments aimed to increase the number of available single-copy genes. As a result, we constructed a new phylogenetic tree (Fig. S3, see online supplementary material) using a smaller population (17 species) but a larger set of single-copy genes (1301). Notably, the new phylogeny provided even stronger support (BS = 100) for the conclusion that Saxifragales is more closely related to the core rosids.

### Comparative analysis and functional prediction of CYPs

CYP superfamily is known for its versatility in catalyzing various biochemical reactions, including the biosynthesis of a wide range of compounds such as plant hormones, flavonoids, coumarins, sterols, carotenoids, amino acids, fatty acids, phenylpropanoids, terpenoids, and alkaloids [16, 27]. To classify the CYP genes in the PC genome, we referred to previous research reports [17] and selected 62 representative CYP genes as a reference (Fig. S4A and B, see online supplementary material).

**Figure 3:**
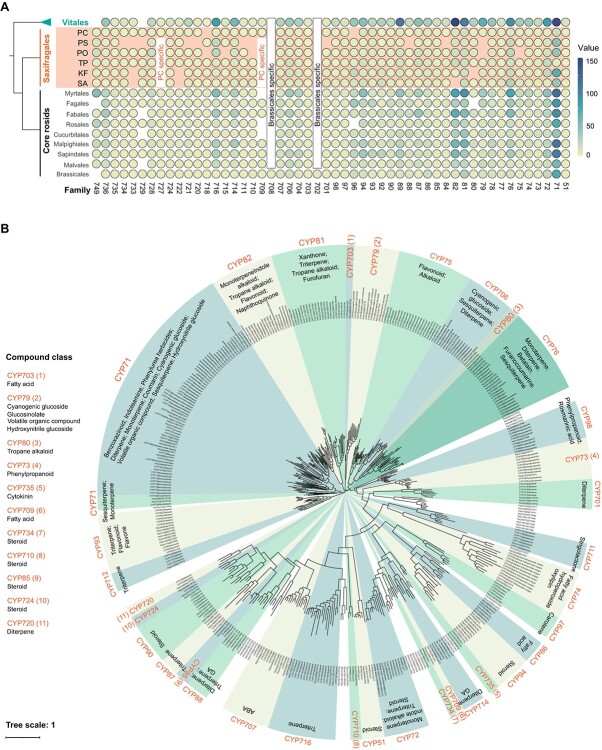
Scanning of cytochrome P450 (CYP) enzymes in the genome of *Penthorum chinense* Pursh (PC). **A** Compare the distribution characteristics of CYP families in Saxifragales, Vitales, and the core rosids within Superrosids. **B** The functional predictions of CYP genes of PC based on a phylogenetic tree constructed using functionally studied CYP genes. KF, *Kalanchoë fedtschenkoi*; PO, *P. ostii*; PS, *Paeonia suffruticosa*; SA, *Sedum album*; TP, *Tiarella polyphylla*.

The PC genome consists of 170 CYP genes, out of which 163 were successfully classified into 57 families using identity values and phylogenetic relationships (Fig. S4C, see online supplementary material), and the remaining seven, which could not be classified due to their short length, were discarded. Among the six species in Saxifragales, the distribution of the CYP families displays certain characteristics, such as the absence of the CYP710 and CYP79 families in *Paeonia*. Additionally, a distinct feature is that the CYP727 and CYP709 families are only present in PC. The distribution of CYP families in Superrosids revealed that two families, CYP729 and CYP749, were absent in Vitales but present in both Saxifragales and the core rosids (Fig. 3A). Interestingly, we did not find any family that was exclusively present in Saxifragales and absent from Vitales and the core rosids, or exclusively present in the core rosids and absent from Saxifragales and Vitales, which potentially supported that Saxifragales is more closely related to the core rosids in evolution. Within the core rosids, some orders have also experienced the losses of the CYP729 and CYP749 families. We speculate that the losses occurred after the divergence of Saxifragales and the core rosids.

Using a large number of functionally studied CYP genes, we predicted the compound classes in which CYP genes participate in synthesis in PC through phylogenetic tree (Fig. 3B), and an insight was gained into the potential metabolic diversity of PC. A total of 35 CYP families have been predicted, many of which are involved in the synthesis of a wide variety of compound classes, such as the CYP71 family, the largest family in the three subclades, that is involved in the synthesis of benzoxazinoids, indoleamines, diterpenes, coumarins, monoterpenes, etc. Additionally, there were many different families involved in the same compound class, such as the families CYP734, CYP710, CYP85, and CYP74, which were all involved in the synthesis of steroids. From an overall perspective, the CYP genes present in PC are involved in the synthesis of a wide variety of metabolites. This suggests that there may be additional and significant pharmaceutical value to be further explored in PC. In the above two PC-specific families in the Saxifragales, CYP709 is mainly associated with fatty acid synthesis. This suggests that there may be certain specific fatty acid-like metabolites in Saxifragales, which might play an important role in metabolic pathways and stress response in PC.

### Synthesis studies of the three most noteworthy medicinal ingredients

**Figure 4:**
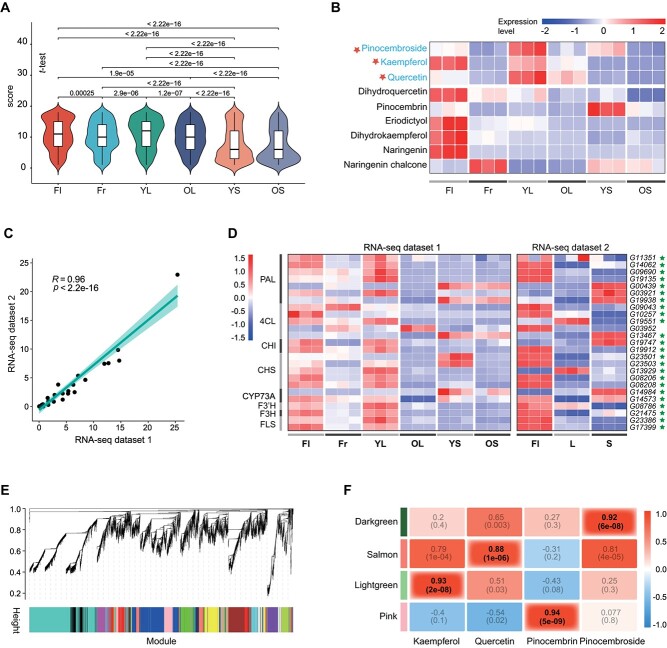
Combined analysis of the transcriptome and flavonoid metabolome based on the *Penthorum chinense* Pursh (PC) genome was conducted. **A** The distribution characteristics of 417 flavonoid metabolites in different tissues. Fl, flowers; Fr, fruits; OL, old leaves; OS, old stems; YL, young leaves; YS, young stems. **B** Expression of nine key flavonoid metabolites in different tissues of the pathway. **C** Pearson correlation for the maximum expression values of 33 genes corresponding to the eight enzymes in each set of RNA-seq samples. **D** Expressions of 25 genes corresponding to eight key enzymes in the flavonoid synthesis pathway in two sets of RNA-seq datasets 1 and 2. L, leaves; S, stems. **E** Hierarchical clustering presenting 27 modules having coexpressed genes. Each leaflet in the tree corresponds to an individual gene. **F** Module-trait associations based on Pearson correlations.

A total of 417 flavonoid metabolites were detected from 18 samples. Principal component analysis (PCA) indicates good consistency among the three biological replicates within each group of samples (Fig. S5A, see online supplementary material). To observe the distribution characteristics of the flavonoid metabolites in different tissues, a scoring scheme was designed (described in ‘Materials and methods’). Box and violin plots demonstrated that, out of the 417 flavonoid metabolites, the highest number with high expression was found in young leaves (YL), followed by flowers (Fl), fruits (Fr), and old leaves (OL) (Fig. 4A). However, in the case of stems, both old and young, the content of most flavonoid metabolites was relatively low. The content of all flavonoid metabolites in different samples was shown in Fig. S6 (see online supplementary material).

According to the detection results of flavonoid metabolites, the synthetic pathway for the three most noteworthy medicinal components in PC: kaempferol, quercetin, and pinocembroside has been drawn according to the KEGG database (Fig. S7, see online supplementary material). As mentioned above, PC contains a significant amount of kaempferol, quercetin, and pinocembroside, which produce liver-protective effects, antioxidation, and anti-inflammation [10–12]. Eight enzymes involved in converting L-Phenylalanine into kaempferol, quercetin, and pinocembroside are phenylalanine ammonia-lyase (PAL), trans-cinnamate 4-monooxygenase (CYP73A), 4-coumarate—CoA ligase (4CL), chalcone synthase (CHS), chalcone isomerase (CHI), flavanone 3-hydroxylase (F3H), flavonoid 3′-monooxygenase (F3’H), and flavonol synthase (FLS).

After quantifying all the genes of PC using two sets of RNA-seq datasets separately, a Pearson correlation analysis was performed on the maximum expression values of 33 genes corresponding to the eight enzymes in each set of RNA-seq samples, and the correlation coefficient (*R*) reached 0.96 (Fig. 4C). PCA indicates good consistency among the three biological replicates within each group of samples of two sets of RNA-seq datasets (Fig. S5B and C, see online supplementary material). Out of the 33 genes, 25 were found to be transcribed and expressed. From Fig. 4D, it can be observed that the gene expressions in the flowers and young leaves are highly active in RNA-seq dataset 1. This may explain why flowers and young leaves contain higher levels of flavonoid metabolites compared to other tissues. In RNA-seq dataset 2, gene expression in the flower is also very active (Fig. 4D); however, leaf samples (L1 ~ L3) may have been collected from both young and old leaves, making it difficult to obtain more hierarchical expression information. The expression activity of genes in the stem is the lowest, and correspondingly, the number of flavonoid metabolites at high levels is also the least.

Although the synthesis pathways of major flavonoid metabolites were already well understood (Fig. S7, see online supplementary material), the specific genes responsible for determining the variations in content of these metabolites across different tissues remain unknown. To address this gap, we employed WGCNA for further investigation. Consequently, we identified 27 distinct gene modules based on their unique co-expression patterns. These gene modules are visually represented by different colors and are presented as a clustergram (Fig. 4E). The contents of kaempferol, quercetin, pinocembroside, and pinocembrin (the precursor of pinocembroside) in each tissue were utilized as phenotypic data for examining the module-trait correlations.

Out of the 27 coexpressed gene networks, four demonstrate significant correlations (*r*^2^ > 0.85) with the content of the four flavonoid metabolites (Fig. 4F); the dark green module is positively correlated with the pinocembroside content (*r*^2^ = 0.92, *P* = 6e-08), the salmon module is linked to quercetin content (*r*^2^ = 0.88, *P* = 1e-06), the light green module is positively associated with kaempferol content (*r*^2^ = 0.93, *P* = 2e-08), and the pink module is positively correlated with pinocembrin content (*r*^2^ = 0.94, *P* = 5e-09). We conducted an analysis on the degree values of each gene within the associated modules and investigated if these genes exhibited differential expression across various tissues. Subsequently, we selected the differentially expressed genes (DEGs) with the highest degree values as the candidate key genes. Genes belonging to transcription factors, CYP families, and flavonoid synthesis pathways were identified, as well as other genes annotated with the KEGG database (Fig. S8, see online supplementary material).

## Discussion

The genomic research on Saxifragales species has been relatively limited, despite their significance in terms of ornamental, economic, and medicinal value [21, 28–32]. However, we have made significant progress by successfully assembling the genome of PC, making it the first Saxifragales species to have a T2T assembly. This achievement holds great importance, as it will enhance our understanding of the synthesis pathways of medicinal ingredients and enable the breeding of varieties with higher concentrations of desired components in PC. The T2T assembly was successful largely due to the advantageous features of the PC genome, such as its small size, low repeat sequence content, and low heterozygosity, which give it a straightforward structure. PC is widely appreciated for its medicinal benefits, but it is also noted for its quick growth cycle, rapid growth rate, and high biomass production. Considering these factors, PC presents itself as an ideal candidate for studying the synthesis mechanisms of multiple secondary metabolites. Here, two crucial aspects should be discussed and elucidated in the findings of this study: the evolution of Saxifragales and the synthesis of the most noteworthy metabolites of PC.

### More reliable inference indicates that Vitales sister to Saxifragales + core rosids

Previous studies have presented conflicting findings regarding the phylogenetic relationships of the three Superrosids subclades (Fig. 2A). One set of studies suggests that Saxifragales is sister to the Vitales, and together they are sister to the core rosids [20, 21]. Another set of studies proposes that Saxifragales is sister to the Vitales + core rosids [22, 23]. Yet another set suggests that Saxifragales is sister to the core rosids, and together they are sister to the Vitales [24, 25]. However, the used data underlying these conclusions is not sufficient, either due to a limited number of species or genes involved in constructing the phylogenetic tree, or due to the use of plastid genomes for phylogenetic tree construction [20–23]. Recent research has utilized data from numerous single-copy nuclear genes, supporting the topology ‘c’ (Fig. 2A) but still raising concerns about the adequacy of the samples collected from the Vitales and Saxifragales [24].

To address these concerns and enhance the reliability of evolutionary analysis, our study included as many species as possible from the Vitales (five species) and Saxifragales (six species). We constructed two maximum-likelihood phylogenies of Superrosids, considering varying numbers of species and single-copy genes, along with using different outgroup species (*D. carota* and *A. sinensis*). Consistently, our results provided strong support (BS = 93 for *D. carota* and 100 for *A. sinensis*) for a closer relationship between Saxifragales and the core rosids compared to Vitales and the core rosids (Fig. 2; Fig. S3, see online supplementary material). Further progress in understanding the phylogenetic relationship among the three subclades in Superrosids can be achieved by constructing additional genomes of species within the Vitales and Saxifragales. This would enable a more precise analysis in the future.

Furthermore, from the distribution characteristics of the CYP families in the three subclades of Superrosids, we can also find some evidence supporting the closer relationship between Saxifragales and the core rosids. However, it should be emphasized that, besides information regarding genetic sequence variations or gene family retention and loss, other types of information, such as phenotype and karyotype [33], should also be carefully studied. These different types of information can complement each other and help deepen our understanding of the relationships between species.

### Study on flavonoid metabolites in PC

Flavonoids are the main components found in PC’s phytochemical profile, including kaempferol, quercetin, and pinocembroside, which have been shown to have liver-protective, antioxidant, and anti-inflammatory effects [10–12]. This study provides a detailed investigation for the first time on the content differences of over 400 flavonoid metabolites in different tissues of PC, including flowers, fruits, leaves, and stems. Additionally, leaves and stems were further distinguished between old and young. We found that the three medicinal components kaempferol, quercetin, and pinocembroside were highest in the flowers and young leaves of PC, which provide crucial reference for the medicinal use of PC. Interestingly, the types and quantities of flavonoid metabolites in PC fruits are not lower than those in the stems. However, fruits have not been considered as medicinal ingredients for a long time [1]. This study primarily focused on the detection of flavonoid metabolites in PC. However, it is important to note that the CYP superfamily of PC plays a diverse role in synthesizing various types of metabolites. It is possible that other types of metabolites, apart from flavonoids, are also present in abundance in PC. Further research on this subject is warranted.

Detailed inter-tissue flavonoid metabolite profiles also provide important evidence for exploring the genes involved in the synthesis and regulation of specific metabolites. As mentioned earlier, the simple genome, short growth cycle, and large biomass of PC make it an excellent subject for studying the mechanisms related to metabolite synthesis and regulation. This study has identified gene sets associated with the regulation of important metabolites such as kaempferol, quercetin, and pinocembroside using WGCNA. However, we have found that regardless of the metabolite, there are many genes involved in its synthesis regulation. This suggests that the regulation of metabolite synthesis is a complex process, making it very difficult to identify the central regulatory genes. In the later stages, combining other methods, such as population genetics and functional genomics, is necessary to confirm the most central genes regulating each key metabolite.

## Materials and methods

### Plant materials and genome sequencing

Gulin County, located in Sichuan Province, China, is known for its production of PC. To ensure the cleanliness of sequencing materials and minimize the risk of microbial contamination, wild PC seeds were collected and planted in sterilized humus soil. The planting process took place in a controlled environment inside a clean incubator at a temperature of 25°C. When the PC seedlings reached a height of approximately 20 centimeters, leaves were carefully collected from one healthy seedling. These leaves were rinsed briefly with sterile water and then dried by shaking. Finally, the plant material was frozen using liquid nitrogen to preserve it for future utilization.

For long-read genomic sequencing, high-quality genomic DNA was extracted using the SQK-LSK110 Ligation Sequencing Kit (Nanopore, Oxford, UK) according to the manufacturer’s instructions. Large DNA fragments (>20 kb) were selected for sequence library preparation. Subsequently, sequencing was performed on the PromethION sequencing platform from Oxford Nanopore Technologies (ONT). To obtain short-read sequences using the Illumina technology, libraries were constructed with 450-bp insertions following the standard Illumina protocol. Paired-end reads were generated on the Illumina HiSeq platform, with the goal of achieving a genome coverage of approximately 60× for conducting comprehensive genome surveys. Additionally, a Hi-C library was prepared using a modified version of a previously published protocol [34]. The Hi-C library was sequenced on the Illumina HiSeq platform using paired-end 150-bp reads.

### Genome size assessment and assembly

Paired-end Illumina reads were utilized for estimating the PC genome size, heterozygosity, and repeat content using GenomeScope2.0 (*k* = 19) [35]. Next, Nanopore reads were corrected, trimmed, and assembled into contigs using Canu v2.2 [36] with the parameters correctedErrorRate = 0.04 and minReadLength = 2000. The raw contigs were further polished and corrected using PILON v1.24 [37]. To construct chromosomes, the ALLHIC pipeline was employed [38]. In this process, high-quality Hi-C reads were aligned to the draft assembly using BWA v0.7.17, and only uniquely mapped reads were chosen for subsequent analysis (https://github.com/lh3/bwa). Telomeres and centromeres in the PC assembly were detected using the software quarTeT [39]. Telomeres were identified based on the presence of the telomeric repeat sequence ‘TTTAGGG’, while repeated sequence annotation results were utilized for centromere detection.

### Deep TE annotations and coding gene predictions

EDTA [40] is a comprehensive approach that combines eight well-established programs to provide a thorough annotation of TEs. EDTA incorporates several tools for different types of TEs: LTRharvest, LTR_FINDER_parallel, and LTR_retriever are used to identify LTR retrotransposons; Generic Repeat Finder and TIR-Learner are included to detect TIR transposons; HelitronScanner recognizes Helitron transposons. To locate TEs missed by other structure-based programs like SINEs and LINEs, RepeatModeler [41] was employed. Finally, homology-based annotation of fragmented TEs is performed using RepeatMasker (https://repeatmasker.org). In order to enhance the annotations of SINEs and LINEs, EDTA utilizes sequence references from the SINEBase database (https://sines.eimb.ru) through the parameter —curatedlib. To classify and name TE sequences that were not categorized in the EDTA annotation results, the DeepTE tool was further utilized [42].

The *de novo* gene prediction was conducted using BRAKER v3.0.3 [43], which automatically generates comprehensive gene structure models by leveraging the combined evidence from RNA-Seq data and homologous protein sequences. Specifically, BRAKER v3.0.3 employed several underlying components for gene prediction, including GeneMark-ETP v3.10 [44], Augustus v3.5.0 [45], and TSEBRA v1.1.1 [46]. The acquisition of RNA-seq data for gene prediction will be mentioned in the following text. Approximately 18 000 homologous protein sequences were obtained from the genome annotations of four Saxifragales species, including *Kalanchoë fedtschenkoi* [38], *Paeonia suffruticosa* [29], *P. ostii* [39], and *Sedum album* [36].

### Phylogenetic inference

The phylogenetic tree was constructed using maximum likelihood based on the concatenation of single-copy genes from 30 species. There were 29 Superrosids species included, and one outgroup species, *D. carota*, was initially used. However, for increased accuracy, the outgroup species was replaced with *A. sinensis*. To enhance the dataset of single-copy genes and construct a new phylogenetic tree at a smaller scale, some species were eliminated. To estimate single-copy genes, OrthoFinder v2.5.5 was utilized [47]. Subsequently, ML analysis was performed using RAxML v8.2.12 [48]. The analysis involved 1000 rapid bootstrap iterations, while the rest of the parameters were set to default. All genome sequences of the included species can be obtained from the plaBi database (https://plabipd.de) and the Ensembl Plants database (https://plants.ensembl.org).

### Flavonoid metabolite profiling

Eighteen samples were collected from flowers (Fl), fruits (Fr), young leaves (YL), old leaves (OL), young stems (YS), and old stems (OS) of PC. Each sample was obtained by mixing ten plants. Samples for flavonoid metabolite detection were prepared according to a previously reported reference [12]. Freeze-drying of the 18 samples was performed using a Scientz-100F vacuum freeze-drier. Afterwards, the freeze-dried samples were ground into a fine powder using a mixer mill (MM400, Retsch) at 30 Hz for 1.5 minutes. Lyophilized powder weighing 100 mg was dissolved in 1.2 ml of a 70% methanol solution. Vortexing was done every 30 minutes for 30 seconds, totaling six cycles. The resulting samples were then refrigerated overnight at 4°C. The next day, centrifugation was done at 12000 × *g* for 10 minutes, and the supernatants were carefully aspirated. Prior to UPLC-MS/MS analysis, the supernatants were filtered using ANPEL SCAA-104 membranes with a pore size of 0.22 mm (Shanghai, China). To assess the repeatability and stability of the measurement process, three quality control (QC) samples were prepared by pooling equal amounts of all sample extracts. For every three samples analyzed, one QC sample was evaluated. To observe the distribution characteristics of the flavonoid metabolites in different samples, a scoring scheme was designed as follows: for each metabolite, a score of ‘18’ was assigned to the sample with the highest content, while a score of ‘1’ was given to the sample with the lowest content.

### Acquisition of RNA-seq dataset

In order to obtain high-quality annotations of coding genes and analyse the synthesis and regulation mechanisms of flavonoid metabolites, two sets of RNA-seq data were obtained.

RNA-Seq dataset 1

According to the manual instructions, total RNA was extracted from the 18 samples using the RNAprep Pure Plant Kit (Tiangen, Beijing, China). Subsequently, cDNA libraries were created using the NEBNext Ultra RNA Library Prep Kit for Illumina (NEB, Harvard, MA, USA). These libraries were then subjected to paired-end sequencing with 150 base pairs. To ensure the quality of the obtained data for subsequent analysis, the raw data was initially filtered using Fastp v0.23.2 [49] with a quality threshold of *q* > 30.

RNA-Seq dataset 2

This dataset was obtained from the NCBI BioProject PRJNA834646. Specifically, the following accessions were retrieved from the SRA database: SRR19090413 ~ SRR19090421. Here, accessions SRR19090413 ~ SRR19090415 (three biological replicates) were collected from flowers (Fl), SRR19090416 ~ SRR19090418 were collected from leaves (L), SRR19090419 ~ SRR19090421 were collected from stems (S).

### Identifications of differentially expressed genes and correlated candidate genes

In the current study, HISAT2 [50] was employed as the aligner to align the sequencing reads. Gene expression quantification to transcripts per million (TPM) was performed using featureCounts [51]. For the analysis of DEGs, we employed DESeq2 [52]. To be considered differentially expressed, genes had to meet the following screening criteria: |log2(fold-change)| >1.5 and an adjusted *P*-value <0.05. WGCNA was performed in R with default parameters to group genes into co-expressed modules. An adjacency matrix was created based on the gene expression data. Following this, the flavonoid metabolite content data was introduced into the WGCNA package, and associations between contents and gene modules were determined using correlation-based calculations, employing the default settings.

### Sequence retrieval and identification of CYP genes

Two methods were employed to identify potential members of the CYP superfamily. The first utilized the Hidden Markov Model (HMM) profile of the P450 domain (PF00067) obtained from the Pfam database to search for candidate CYP proteins [53]. The second involved using BLASTp, a sequence similarity search tool. For constructing the BLAST database, reference CYP proteins were selected from several species: *Brassica rapa* and *Arabidopsis thaliana* (belonging to the Brassicaceae family), *Oryza sativa* (Poaceae family), *Populus trichocarpa* (Salicaceae family), *Chlorella vulgaris*, and *Physcomitrella patens* (both Chlorophyta species). These species were chosen because they possess CYP genes covering all 11 CYP clans found in land plants [17]. The obtained potential CYP members were then subjected to a P450 domain search using CD-Search [54], and sequences without the P450 domain were removed.

## Supplementary Material

Web_Material_uhad274

## Data Availability

The genome raw sequencing data, Hi-C data, and transcriptome raw sequencing data have been deposited at the NCBI Sequence Read Archive under BioProject number PRJNA1063843.
