# Supplementary material for: Insights into the Superrosids phylogeny and flavonoid synthesis from the telomere-to-telomere gap-free genome assembly of Penthorum chinense Pursh
Source: Hortic Res. 2023 Dec 19;11(2):uhad274. doi: 10.1093/hr/uhad274 (PMC10857932; doi:10.1093/hr/uhad274)
Supplement: Web_Material_uhad274 [file web_material_uhad274.zip › Supplemental Data Figures.docx]

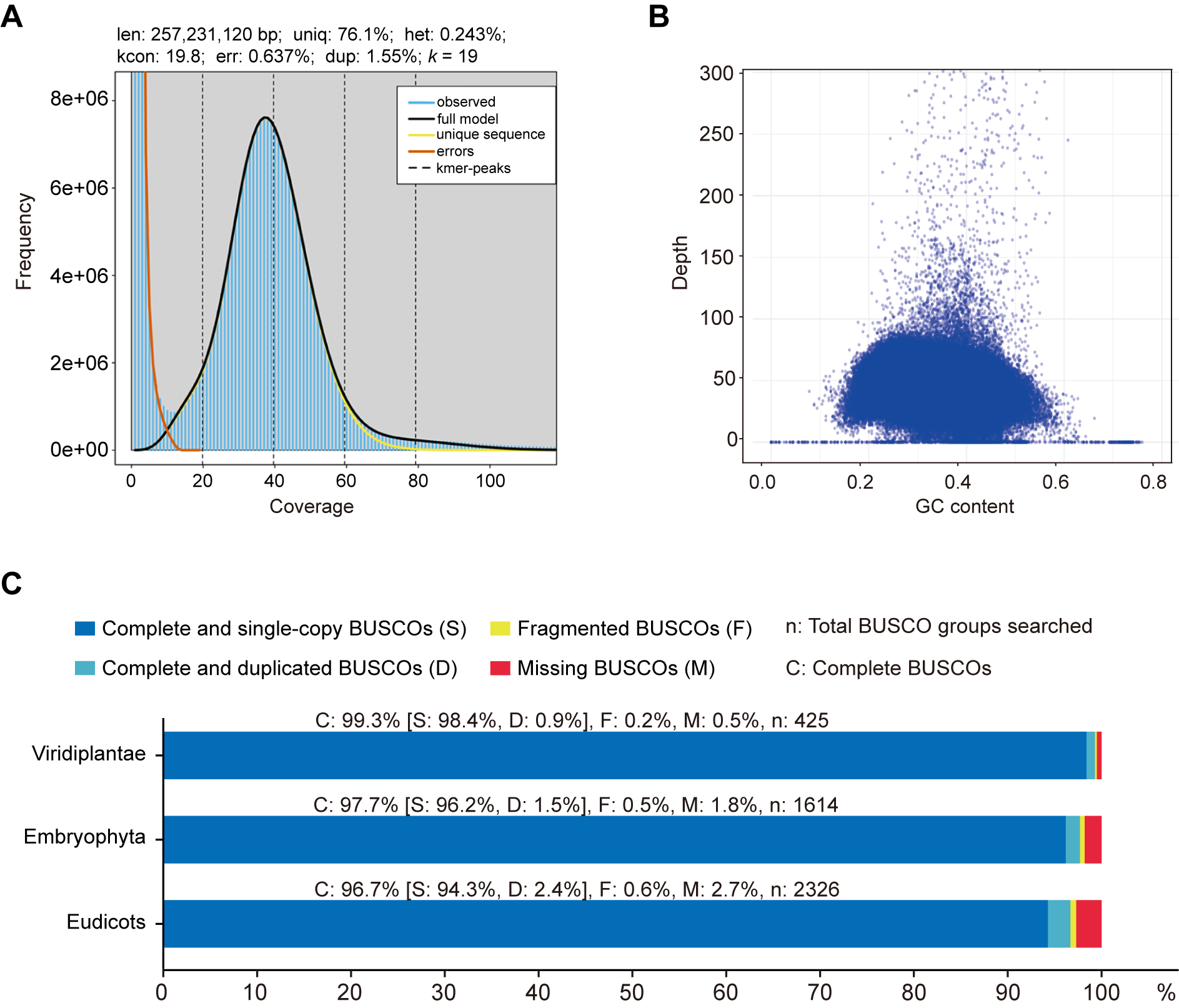


**Supplementary Data Figure S1.** Genome evaluation and assembly. **A** The genome of *Penthorum chinense* Pursh was estimated to be 257.2 Mb, with a low heterozygosity of 0.24%, based on *k*-mer frequencies (*k* = 19) of Illumina short reads. **B** The depth graph of the GC content indicates that the assembly is free from contamination by other species. **C** The high complete Benchmarking Universal Single-Copy Orthologs (BUSCO) coverage values assessed using three databases confirm the accuracy and integrity of the gene annotations.


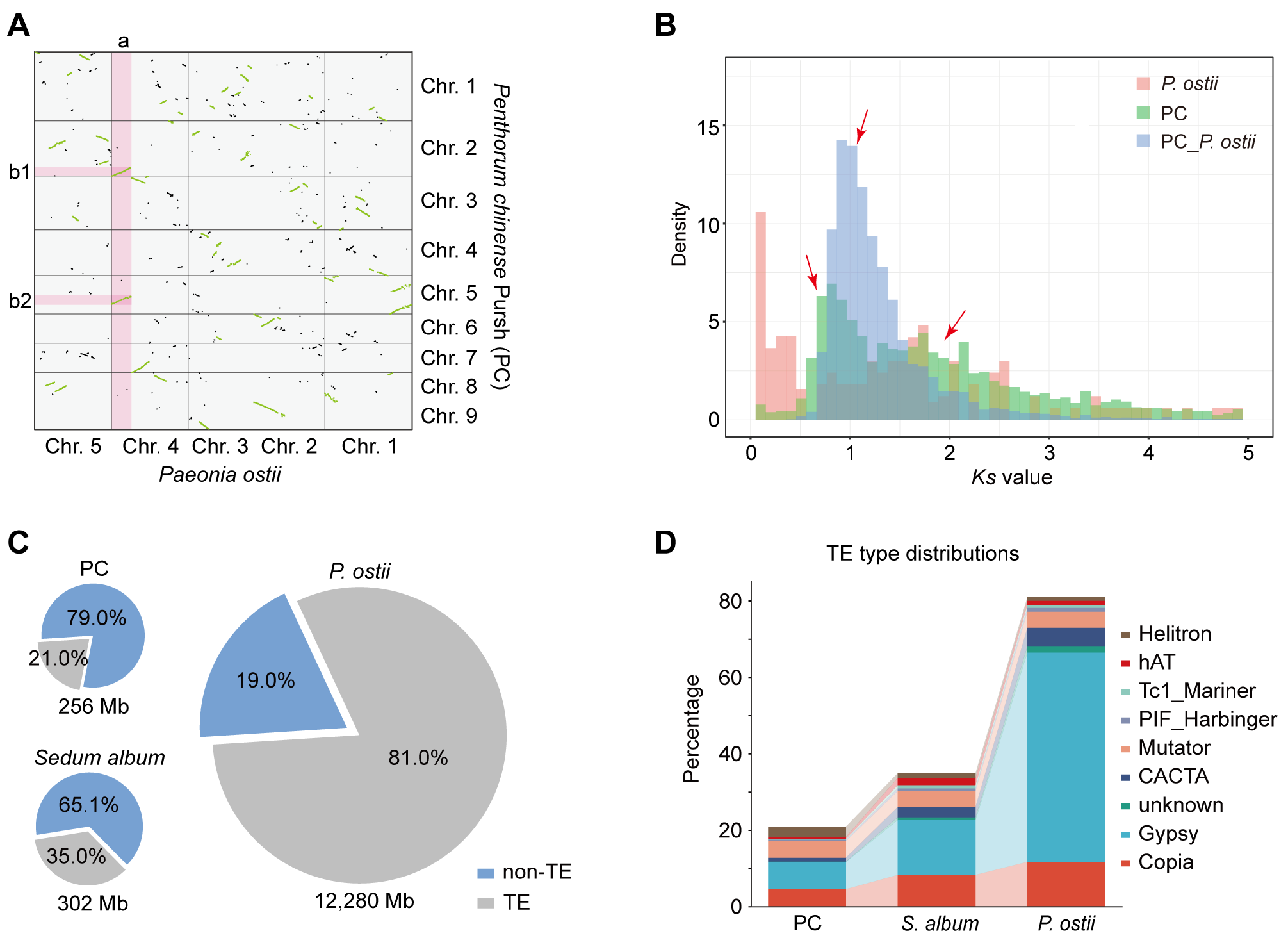


**Supplementary Figure S2.** Investigating the reason for the much smaller genome size of *Penthorum chinense* Pursh (PC) compared to that of *Paeonia ostii*. **A** Collinearity analysis found that genome fragment “a” (Chr. 4) of *P. ostii* corresponds to two genome fragments “b1” (Chr. 2) and “b2” (Chr. 5) of PC. **B** Ks distribution indicates that after species differentiation from *P. ostii*, a separate whole genome replication event was conducted for the PC genome. **C** The genome of *P. ostii* is much larger than those of PC and *Sedum album*, and the proportion of transposable elements (TEs) in the *P. ostii* genome is also significantly higher than in theirs. **D** The proportion of TEs in the genomes of the three species PC, *P. ostii* and *S. album.*


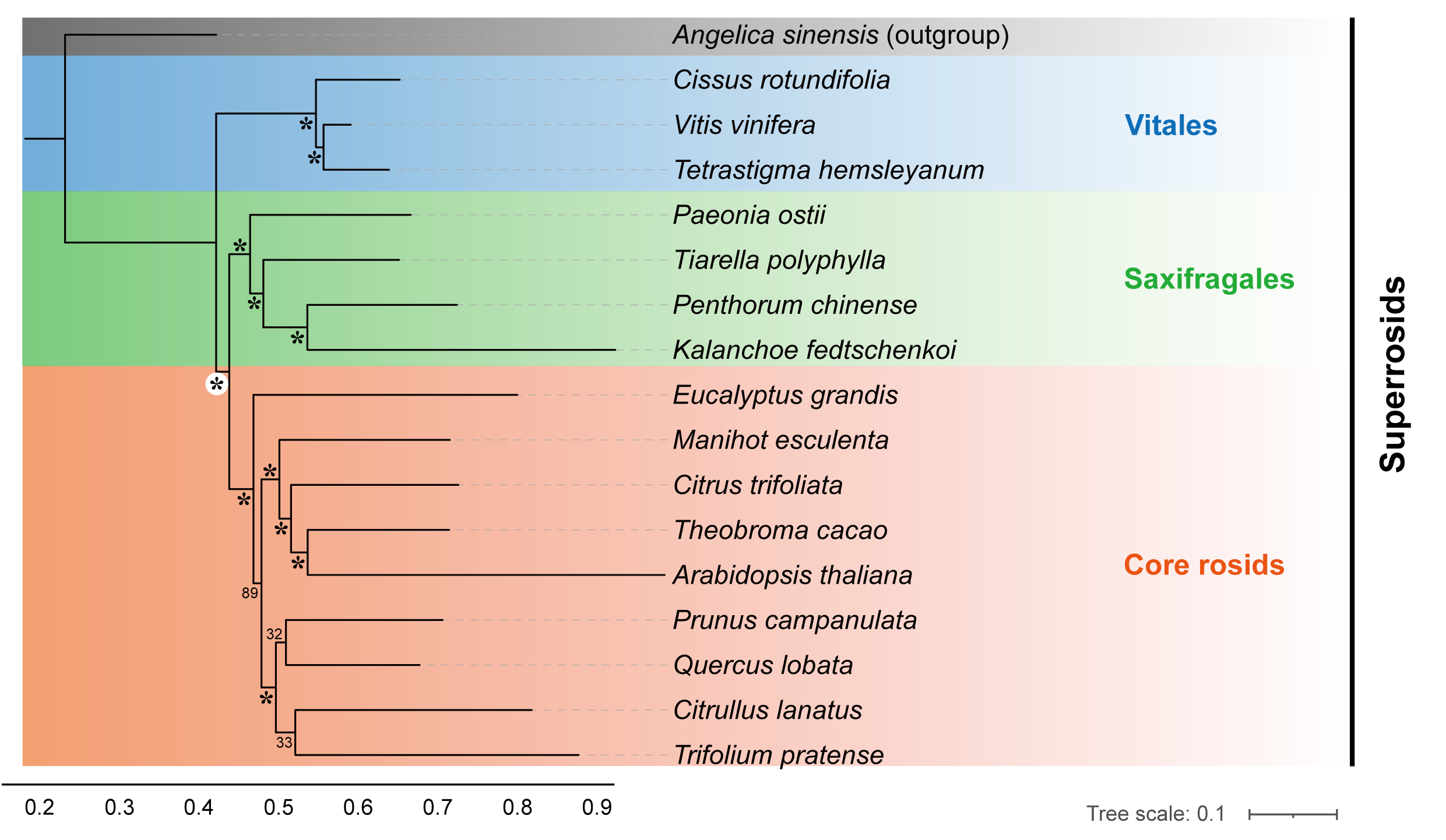
 **Supplementary Data Figure S3.** The phylogenetic relationships of the Superrosids species were ensured to be accurate by replacing the outgroup species with *Angelica sinensis* and removing some species from Vitales and Saxifragales that belonged to the same genus. We also eliminated species from the core rosids clade belonging to the same order. This approach aimed to increase the number of single-copy genes and create a new phylogenetic tree using a smaller population (17 species) and a larger set of genes (1,301 single-copy genes). The asterisk (*) indicates that the clade is supported by a BS value of 100.


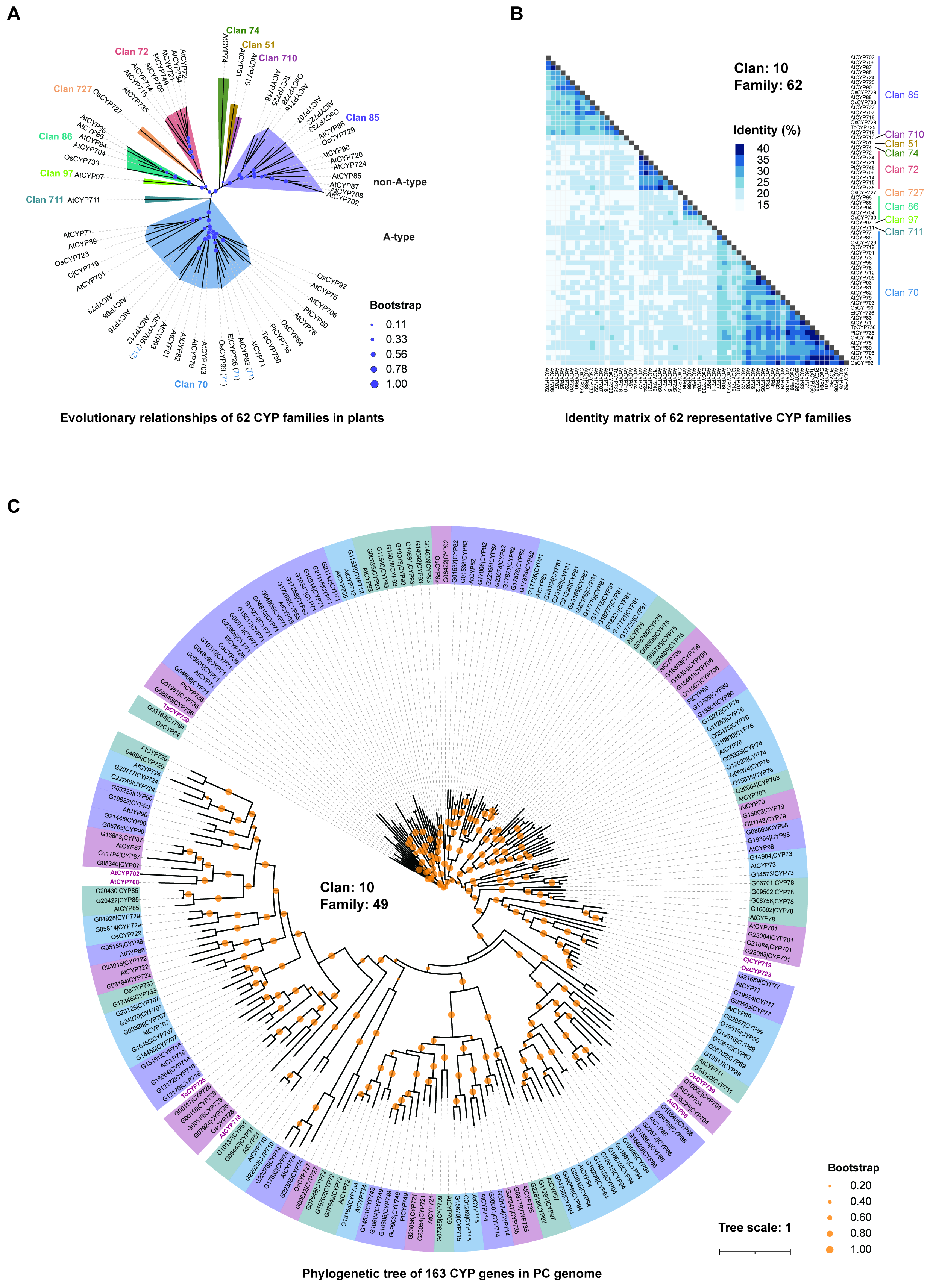


**Supplementary Data Figure S4.** Identification of the cytochrome P450 (CYP) family in *Penthorum chinense* Pursh (PC). **A** The phylogenetic relationships of 62 representative CYP families from 10 clans, including genes from *Arabidopsis thaliana* (At), *Oryza sativa* (Os), *Brassica rapa* (Br), *Thellungiella parvula* (Tp), *Populus trichocarpa* (Pt), *Coptis japonica* (Cj), *Taxus cuspidata* (Tc) and *Euphorbia lagascae* (El) have been studied. **B** A identity matrix of 62 representative CYP genes. **C** The phylogenetic tree of the 163 CYP genes from 57 families and 10 clans of the PC genome.


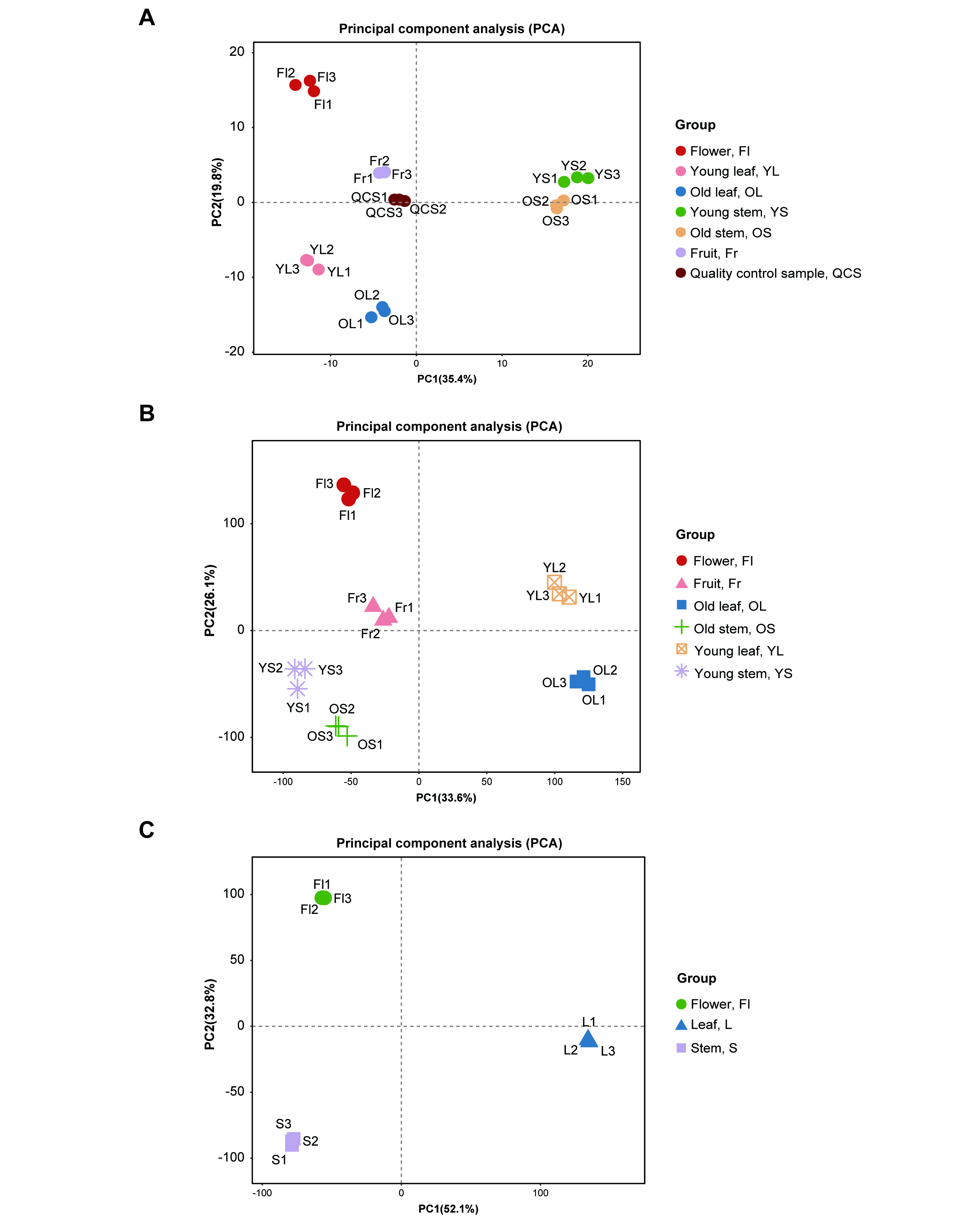


**Supplementary Data Figure S5.** Principal component analysis (PCA). **A** The metabolite detection result demonstrates the biological replicates form each group (dataset 1) exhibit excellent reproducibility. **B** The transcriptome quantification also shows that the biological replicates from each group (dataset 1) are highly reproducible. **C** Similarly, the biological replicates of each group from the dataset 2 obtained from the NCBI BioProject PRJNA834646 exhibit excellent consistency.


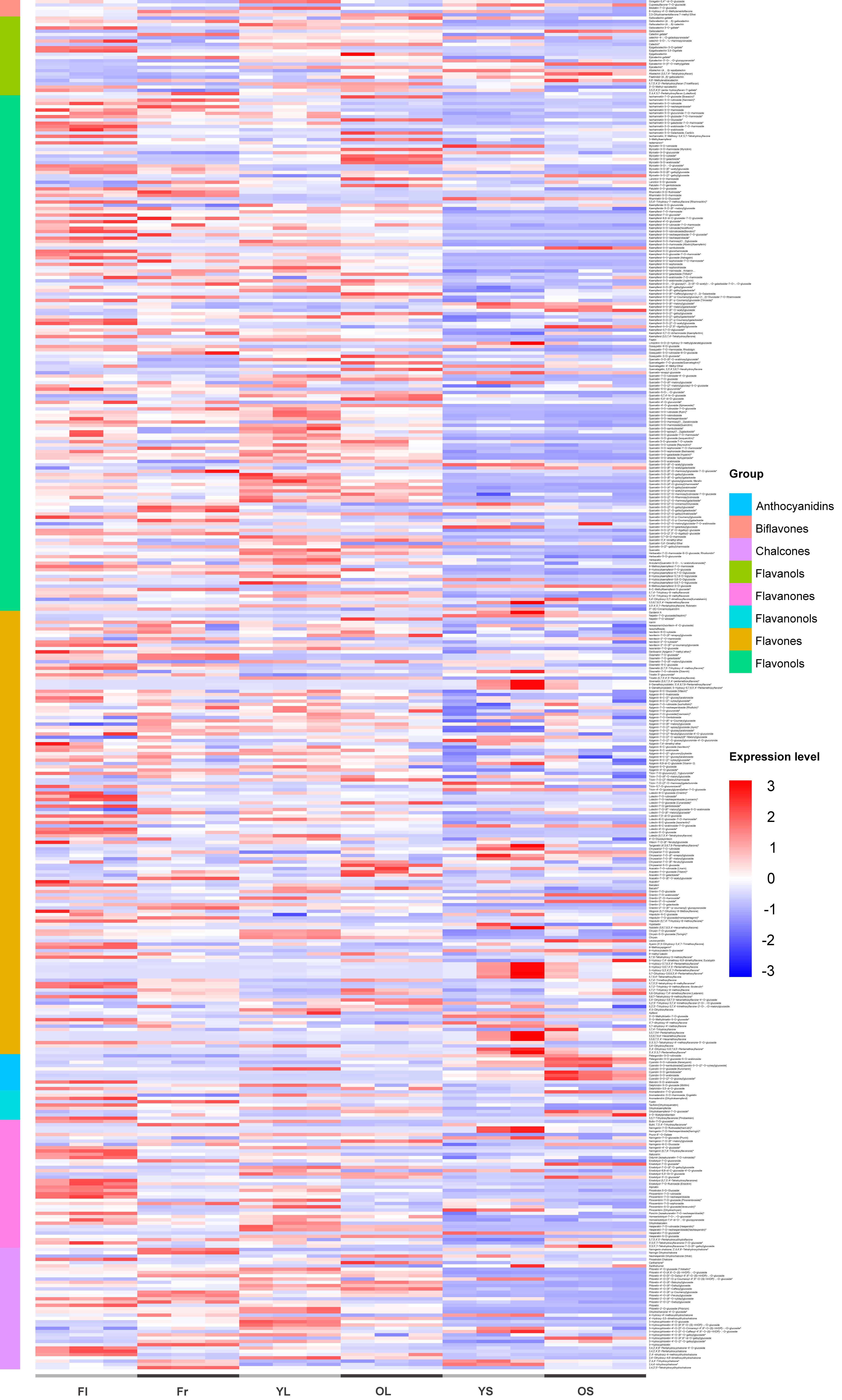


**Supplementary Data Figure S6.** The heatmap of the distribution characteristics of 417 flavonoid metabolites in different tissues of *Penthorum chinense* Pursh. Fl, flowers; Fr, fruits; YL, young leaves; OL, old leaves; YS, young stems; OS, old stems (OS).


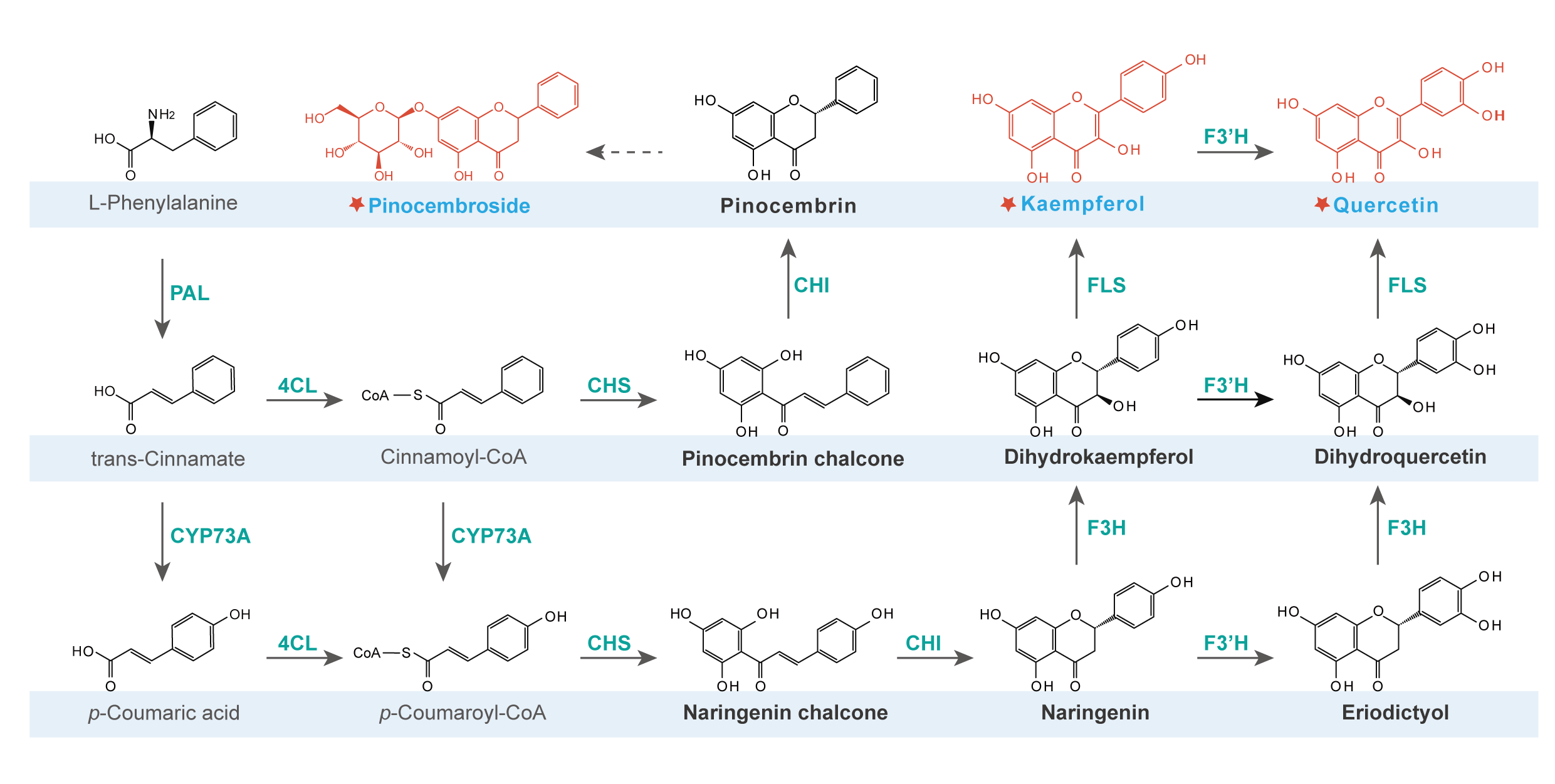


**Supplementary Data Figure S7.** The synthesis pathway of the three most noteworthy medicinal components (kaempferol, quercetin, and pinocembroside) of *Penthorum chinense* Pursh. Eight key genes (marked in cyan) were annotated, including phenylalanine ammonia-lyase (PAL), trans-cinnamate 4-monooxygenase (CYP73A), 4-coumarate-CoA ligase (4CL), chalcone synthase (CHS), chalcone isomerase (CHI), flavanone 3-hydroxylase (F3H), flavonoid 3'-monooxygenase (F3’H), and flavonol synthase (FLS).


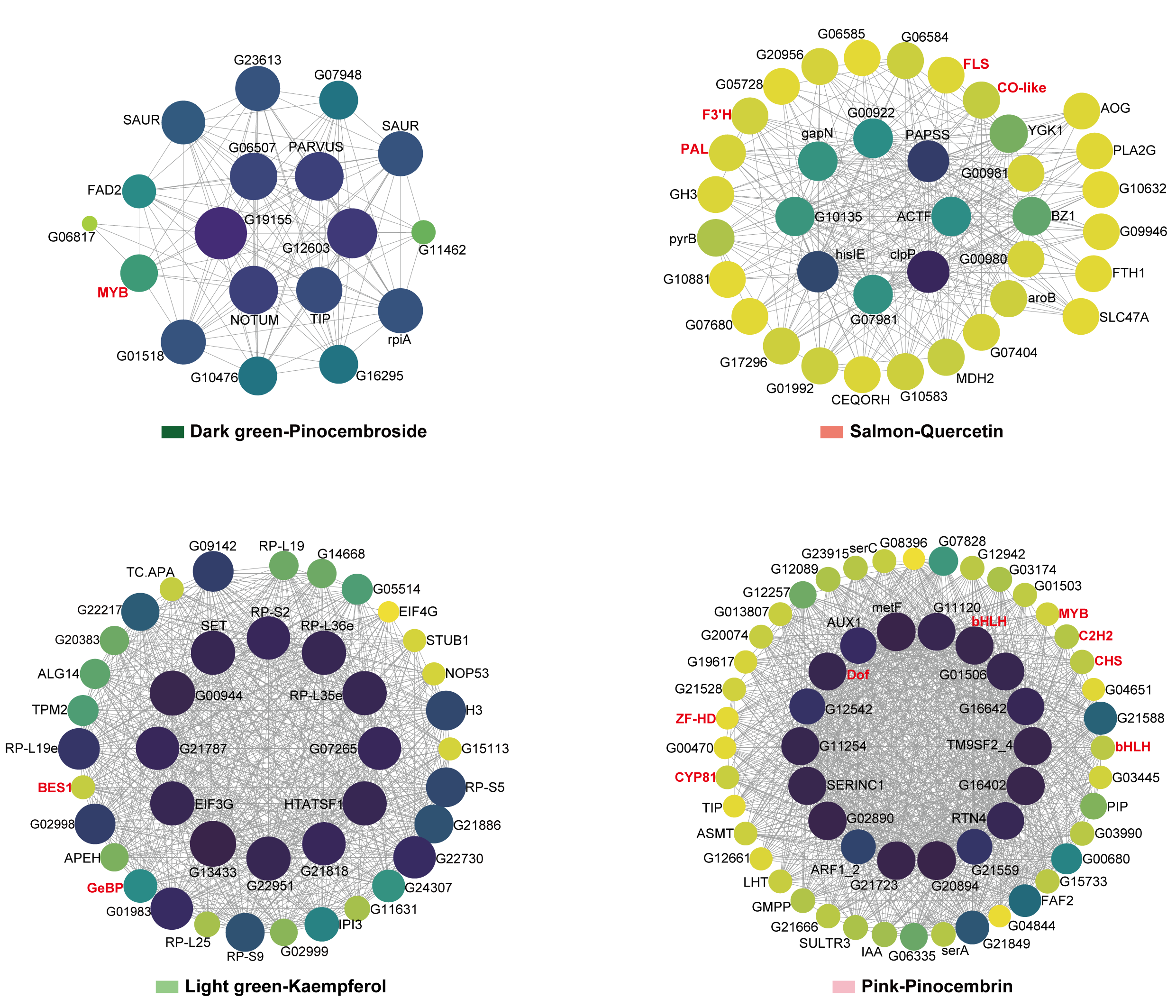


**Supplementary Data Figure S8.** Gene regulatory networks related to the synthesis of metabolites kaempferol (light green), quercetin (salmon), pinocembroside (dark green) and pinocembrin (pink) are presented. The differentially expressed genes with the highest degree values were identified as the candidate key genes. Genes belonging to transcription factors, cytochrome P450 (CYP) families, and flavonoid synthesis pathways were identified, as well as other genes annotated with the KEGG database.
