# Supplementary material for: Insights into the Superrosids phylogeny and flavonoid synthesis from the telomere-to-telomere gap-free genome assembly of Penthorum chinense Pursh
Source: Hortic Res. 2023 Dec 19;11(2):uhad274. doi: 10.1093/hr/uhad274 (PMC10857932; doi:10.1093/hr/uhad274)
Supplement: Web_Material_uhad274 [file web_material_uhad274.zip › Supplemental Data Tables.docx]

| Target sample (TS) | Reference species (RS) | Fluorescence  intensity of RS | Fluorescence  intensity of TS | Ratio | Genome size (Mb) |
| --- | --- | --- | --- | --- | --- |
| Biological repeat-1 | *Lycopersicum esculentum* | 17.66 | 5.36 | 0.3035 | 267.1 |
| Biological repeat-2 | *Oryza sativa* | 8.96 | 5.75 | 0.6417 | 256.7 |
| Biological repeat-3 | *Oryza sativa* | 7.96 | 5.22 | 0.6558 | 262.3 |
| Biological repeat-4 | *Oryza sativa* | 8.16 | 5.47 | 0.6703 | 268.1 |
| Average | / | 10.69 | 5.45 | 0.5678 | 263.6 |

**Supplementary Data Table S1.** Genome size of *Penthorum chinense* Pursh assessed using flow cytometry

**Supplementary Data Table S2.** Comparative analysis of the transposable element content of three representative Saxifragales species *Penthorum chinense* Pursh, *Sedum album*, and *Paeonia ostia*

| Order | Superfamily | *P. chinense* Pursh (Mb/%) | *S. album* (Mb/%) | *P. ostii* (Mb/%) |
| --- | --- | --- | --- | --- |
| LTR | Copia | 11.9/4.61 | 25.2/8.35 | 1,446.7/11.78 |
|  | Gypsy | 18.5/7.17 | 43.3/14.34 | 6,720.0/54.74 |
|  | unknown | 0.1/0.05 | 2.2/0.73 | 194.0/1.58 |
| TIR | CACTA | 2.7/1.03 | 8.3/2.74 | 604.5/4.92 |
|  | Mutator | 11.1/4.31 | 12.7/4.19 | 517.5/4.21 |
|  | PIF_Harbinger | 1.1/0.43 | 1.9/0.64 | 121.0/0.99 |
|  | Tc1_Mariner | 0.5/0.20 | 2.6/0.85 | 97.1/0.79 |
|  | hAT | 1.2/0.47 | 5.7/1.87 | 132.0/1.07 |
| non-TIR | helitron | 7.0/2.70 | 3.8/1.25 | 110.0/0.90 |
| Total | / | 54.0/20.98 | 105.7/34.95 | 9,942.8/81.0 |
